# Shotguns vs Lasers: Identifying barriers and facilitators to scaling-up plant molecular farming for high-value health products

**DOI:** 10.1371/journal.pone.0229952

**Published:** 2020-03-20

**Authors:** Jonathan Menary, Matthew Hobbs, Sara Mesquita de Albuquerque, Agata Pacho, Pascal M. W. Drake, Alison Prendiville, Julian K-C. Ma, Sebastian S. Fuller

**Affiliations:** 1 Institute for Infection and Immunity, St George’s University of London, Tooting, London, United Kingdom; 2 London College of Communication, University of the Arts, London, United Kingdom; Arizona State University, UNITED STATES

## Abstract

Plant molecular farming (PMF) is a convenient and cost-effective way to produce high-value recombinant proteins that can be used in the production of a range of health products, from pharmaceutical therapeutics to cosmetic products. New plant breeding techniques (NPBTs) provide a means to enhance PMF systems more quickly and with greater precision than ever before. However, the feasibility, regulatory standing and social acceptability of both PMF and NPBTs are in question. This paper explores the perceptions of key stakeholders on two European Union (EU) Horizon 2020 programmes–*Pharma-Factory* and *Newcotiana*–towards the barriers and facilitators of PMF and NPBTs in Europe. One-on-one qualitative interviews were undertaken with N = 20 individuals involved in one or both of the two projects at 16 institutions in seven countries (Belgium, France, Germany, Italy, Israel, Spain and the UK). The findings indicate that the current EU regulatory environment and the perception of the public towards biotechnology are seen as the main barriers to scaling-up PMF and NPBTs. Competition from existing systems and the lack of plant-specific regulations likewise present challenges for PMF developing beyond its current niche. However, respondents felt that the communication of the benefits and purpose of NPBT PMF could provide a platform for improving the social acceptance of genetic modification. The importance of the media in this process was highlighted. This article also uses the multi-level perspective to explore the ways in which NPBTs are being legitimated by interested parties and the systemic factors that have shaped and are continuing to shape the development of PMF in Europe.

## Introduction

Plants offer a convenient and cost-effective expression system for the production of high-value recombinant proteins [1]. Plant molecular farming (PMF) has been used to produce monoclonal antibodies the targeting HIV, Rabies and Ebola viruses [2–5]. PMF can also been used for the production of nanoparticles for biomedical use [6,7] and compounds for cosmetic purposes [8–10]. These are often referred to as plant-made (or plant-derived) pharmaceuticals, plant-made industrials, biopharmaceuticals or biologics. For simplicity, the term plant-made pharmaceuticals (PMPs) is used in this article.

The demand for such high-value molecules has grown and continues to rise, sometimes outstripping supply from existing protein expression systems that rely on yeast, bacterial and mammalian cell cultures [11]. Plants have several advantages over these protein expression systems, namely: (i) lower production costs; (ii) low risk of contamination with human pathogens; (iii) scalability of cultivation; (iv) and expertise and infrastructure in place for the production of plant material [9,12–15].

At the same time, recent advances in biotechnology are finding plant breeding applications. These are collectively known as new plant breeding techniques (NPBTs) and can be used to produce improved crop varieties that would be difficult to obtain by using traditional breeding methods or transgenic modification [16,17]. The CRISPR-Cas9 system, for example, has already been used to improve the efficacy of *Nicotiana tabacum* and *N*. *benthamiana* as PMF platforms [18,19], with CRISPR offering the possibility for rapid development of plant-based protein expression using generic recipient lines for desired recombinant DNA [20].

Despite the potential of PMF to impact global health [21] and NPBTs to improve PMF expression systems, previous research has identified several non-technical barriers to the development of these technologies.

### Newcotiana & Pharma-Factory

Two separate but complimentary Horizon 2020 projects include work packages to explore the social, economic and environmental issues associated with PMF and NPBTs in the EU and contribute to the responsible development of these innovations.

The Pharma-Factory project (https://pharmafactory.org) is coordinated by St George’s University of London and involves 14 institutions (13 in EU member states and one in Israel). The project aims to resolve the technical, social and economic bottlenecks for PMF. It takes a product-led approach with five small to medium enterprises (SMEs) involved in the production of medical, veterinary and diagnostic products—it also aims to involve all relevant stakeholders in the process of developing these platforms, including scientists, representatives of PMF businesses and the public at large. The public engagement work-package of the Pharma-Factory project places particular emphasis on facilitating communication between these groups [22], public engagement and understanding barriers to social acceptance.

The Newcotiana project (https://newcotiana.org) is coordinated by Consejo Superior de Investigaciones Científicas (CSIC) in Spain and involves 19 institutions (18 in EU member states and one in Australia). The programme of work focuses on the application of NPBTs to improving the tobacco plant (*N*. *tabacum)* and its relative (*N*. *benthamiana)* as PMF platforms. It is hoped that these efforts will lead to the tobacco plant becoming a model crop for a variety of PMP targets, which would in turn lead to shorter development times for new products and testing. The public engagement work-package of the Newcotiana project aims to engage the public as well as relevant stakeholders to determine barriers and facilitators of PMF, with a particular emphasis on understanding attitudes towards NPBTs.

To provide context for these work packages, scoping research was undertaken to develop an understanding of the perceived barriers and facilitators of PMF amongst key stakeholders (i.e. those involved in research and development of PMF platforms). Future work expands on this approach by involving wider groups of stakeholders and the public.

### Plant molecular farming

The use of plants as platforms for the expression of recombinant proteins was first demonstrated in the late 1980s and early 1990s –primarily with the tobacco plant [23–25]. It would take over a decade for the first commercial product to be launched [8], but since that time a large number of products have been commercialised or are in development [9,26]. Today, PMF relies on a number of methods for protein expression: (i) stable nuclear transformation of a crop species that will be grown in a field or glasshouse; (ii) stable plastid transformation of a crop species; (iii) transient transformation of a crop species in containment; and (iv) stable transformation of a plant species that is grown hydroponically such that the protein is secreted into the medium and recovered [12]. Each method has its strengths and weaknesses, depending on target molecule. The short timescales required to produce large amounts of protein via transient expression makes it suitable for products with irregular demand or unstable markets [9,27]. The stable transformation of crop species takes much more time, but represents the most scalable production method [9]–and potentially the most controversial from a social perspective. PMF represents the third ‘generation’ of genetically modified (GM) plants: first-generation crops were bred for productivity, second-generation crops for food quality and third-generation crops for the production of PMPs and industrial purposes [28,29].

Four factors differentiate PMF from the older, first-generation biotechnology paradigm:

1. Purpose: the aim of PMF differs from the plant breeding of food and feed crops for direct consumption by humans or animals [30].
2. Non-food status of (some) PMF platforms: the use of non-food/feed crops such as tobacco may limit the risk of gene transfer to food crops [31] and reduce public concern over contamination [30,32].
3. Transient expression: proteins of interest are extracted without modifying the germline of plant (instead only modifying the bacteria that infects the plant). These molecules are produced in contained facilities, which may reduce concerns over contamination [31,33].
4. New plant breeding techniques: NPBTs differ from first-generation transgenic breeding techniques in terms of precision and, in some cases, do not result in products that contain transgenic material [16,34].

Despite these factors, PMF is still in the process of gaining the social acceptability currently enjoyed by rival expression systems [11].

### New plant breeding techniques

New plant breeding techniques are a collection of specific biotechnologies that have largely emerged over the last two decades [35]. These include site-specific nuclease (SSN) technologies, such as the CRISPR-Cas9 and zinc finger nucleases (ZFNs), so named due to their ability to modify target genomes at designated points. These techniques are also known as gene editing/engineering and permit *cisgenesis* (breeding plants using only genetic material native to that species or from a sexually compatible relative) and *intragenesis* (using only genes native to a particular species or close relative, but in novel combinations).

Whilst NPBTs often rely on an initial transgenic “step”, these techniques can produce plants with no final transgenic material that are indistinguishable from those bred by conventional breeding approaches [16,34,36,37]. Crucially, gene editing is viewed by experts as a significant departure from earlier plant biotechnology because it permits the inactivation of endogenous genes, precise conversion of alleles and the insertion of whole genetic elements from close relatives [38]. As such, NPBTs have renewed the debate about how to regulate biotechnology by creating a ‘grey area’ for plant breeding legislation [22].

In the EU, NPBTs have fallen under the umbrella of existing (first-generation, transgenic) GM legislation (2001/18/EC) [39,40]; the decision of the European Court of Justice (ECJ) in 2018 that directed mutagenesis is covered by this regulation has caused concern amongst the European scientific community over the impact to biotechnology investment and commercialisation prospects [41,42]. In the United States and other countries in the Americas, crops bred with NPBTs have found relatively quick routes to market where regulation is no stricter than for conventionally-bred crops [43]. These have included crops with targeted gene ‘knockouts’, such as a non-browning mushroom with reduced polyphenol oxidase production [44]. The alternate regulatory ‘triggers’ in the EU and in the USA represent the two broad pathways to regulating modified crops [39]: *process-* and *product*-based regulation. A process-based regulatory trigger responds to crops produced by specific technologies, as in the EU [39]. A product-based regulatory trigger responds to products with specific, often “novel” characteristics, such as transgenes. In practice, the two pathways are both subject to considerable local variations and exemptions, which Eckerstorfer et al. [39] argue will prevent international harmonisation in the near future.

The regulatory debate intersects the wider issue of social acceptability and whether the regulatory approach to NPBTs should address public concerns over the risks of biotechnology or, where these are considered to be divergent, be based purely on known environmental risks of such biotechnology [45,46]. Malyska et al. [46] argue that EU legislation on the cultivation of GM crops was driven largely by public concerns about risk [see also 47], rather than by the interpretation of scientific evidence. Various theories have been proposed to explain public reaction to biotechnology in Europe, such as the ‘knowledge deficit’ model, which suggests that it is a lack of knowledge about science leads to resistance against it. Some authors have argued that a lack of sufficient information about biotechnology has led to consumers relying on the advice of ‘other’ stakeholders such as consumer and environmental organisations in the past [47]. However, this model is not without its criticisms [48,49]. Some studies have found no association between information deficits and acceptance [50]. Others have found that social trust is more important than knowledge with respect to public perceptions of new technology [51]. Besley et al. [52], for example, show an increase in negative attitudes towards scientific studies that include large commercial partners.

Certain EU directives (2005/412) have meant that member states can unilaterally ‘opt-out’ of GM crop cultivation [53]. The ECJ’s 2018 ruling on NPBTs could lead to member states employing this opt-out for NPBT-bred crops in the future, with implications for PMF. Yet questions remain over the social acceptability of PMPs from NPBT plants: do consumers know or care how these are derived? Ultimately, whilst there has been a substantial academic effort to understand stakeholder and public attitudes towards first-generation GM food and feed crops, much less has been done with regards to PMF—and less still at the intersection of PMF and NPBTs, which has only emerged as a technological pairing in recent years.

### The multi-level perspective

As NPBTs test existing European regulation on GM crops, PMF tests regulation that concerns the production of pharmaceuticals [54,55]. Some commentators have argued that PMPs are constrained by existing regulation designed for cell culture technology that does not account for the distinct advantages of plant-based expression systems [56]. Technological trajectories are often restricted by current standards and past choices that in turn result in new technologies being “locked out” of a market, independent of the inherent qualities of the technologies themselves [57–59].

One theoretical framework to understand how new technologies emerge and begin to challenge the existing socio-technical *regime*–in this case, bacterial and mammalian protein expression systems—is the multi-level perspective (MLP). MLP proposes that ‘niches’ represent spaces in which to develop new technology that are isolated in some way from normal market pressures, such as university laboratories [60]. Niche-innovations are often carried and developed by small networks of what Geels & Schot call ‘dedicated actors’ [61], of which the PMF community makes an interesting case study. An example of this is the ZMapp Ebola virus vaccine (Mapp Biopharmaceutical), developed in tobacco (*N*. *benthamiana*) to respond to the 2014 Ebola Crisis in West Africa [4]; the lack of cell culture alternatives and speed of protein acquisition provided an ideal space in which to demonstrate the effectiveness of PMF [62–64].

A further unit of analysis in MLP is the *landscape*; this is the slow-changing, macro-level environment in which regimes sit. Changes in the landscape—such as policies supporting “greener” industrial processes, for example—can exert pressure on the regime, creating opportunities for niche-innovations to gain market share and change existing modes of business.

The somewhat slow development of PMF products since the idea was first proposed in the 1980s may be explained by a lack of landscape-level pressure [64]. Dolfsma & Leydesdorff [59] point out that technologies can break out of such conditions if a ‘third’ (i.e. non-market and non-technological) factor can “unpick” the lock of the existing regime. For PMF, this could be *plant-specific* regulations or protocols. Until such regulation is developed, there will be a disincentive for commercial development of (some) PMPs [56]. One means by which favourable policy can be advanced is through legitimation; this is the process by which actors frame issues in a way that confers legitimacy to their actions or technologies, often by creating positive discourses around their innovation [60]. In the case of PMF, this often relies on comparisons with existing protein expression systems (e.g. plants being cheaper or more scalable) and the opportunities to improve health outcomes in low- and middle-income countries [see 65].

There is a need to understand how these factors could influence future health outcomes. This article describes a qualitative scoping study with key PMF experts, which helped guide the development of follow-on research for Newcotiana and Pharma-Factory projects public engagement work.

The research questions framing this initial scoping study were: what are the barriers and facilitators of PMF and NPBTs in Europe? How are PMF products perceived by experts and how might they be perceived by end-users? How have PMF experts engaged with the public about these technologies?

It is hoped that this study will contribute to ongoing discussions about the future of PMF and emerging debates about the appropriate regulation of NPBTs, as well as provide an initial framework for understanding the development of both technologies through the multi-level perspective.

### Methodology

The methodology of the study involves two overlapping parts of the Pharma-Factory and Newcotiana engagement work. Both studies focused on gaining an understanding of the opinions of stakeholders towards the risks and opportunities of PMF and NPBTs and what might constitute barriers and facilitators to the ongoing development of these technologies. An applied qualitative approach was employed; the need to understand varied social phenomena necessitated an in-depth understanding of the topic. Qualitative research can provide four classifications of information [66]:

1. Contextual: describing what exists
2. Explanatory: examining the reasons for and relationships between what exists
3. Evaluative: appraising the effectiveness of what exists
4. Generative: supporting the development of theories, strategies and actions

Each of these categories is important for understanding the barriers and facilitators for scaling-up PMF. Firstly, a contextual understanding of the practicalities of developing PMF products is required to situate subsequent information. Secondly, explanatory accounts of support for or opposition to genetic modification, for example, are important. The evaluation of how well PMF platforms are performing and the perceived suitability of the regulatory environment in which they currently operate will likewise be valuable. Lastly, generative accounts of possible facilitation mechanisms will provide a basis for future work on the Pharma-Factory and Newcotiana projects.

### Semi-structured interviews with key consortium stakeholders

Semi-structured one-on-one interviews were chosen as the most suitable method of data generation for this study on the basis that they can provide the level of depth required to explore each the categories described above. Semi-structured interviews allow the researcher to probe interesting lines of inquiry during the interview in a flexible manner [67].

An interview guide was developed for each study (available in the project repositories). These were created following consultation with the relevant literature on PMF and NPBTs respectively. An interview guide helps the interviewer structure the interview by orientating it around topics relevant to the study aims [68]. In the case of Newcotiana, the interview guide concerned: 1) the risks and benefits of using NPBTs to modify *Nicotiana* lines 2) personal and public perceptions of NPBTs and 3) participation in public engagement activities. For Pharma-Factory, the interview guide was focused on: 1) perceptions of plant-derived products, 2) key stakeholders and end-users for PMPs and 3) experience with stakeholder and end-user engagement. Information about the participant’s current role, background and research activities was also taken, in order to contextualise their responses.

The sampling frame for this study can be described as purposive [69]. The inclusion criteria were: 1) the individual must have experience in developing PMF platforms and 2) be associated with either the Pharma-Factory and/or Newcotiana projects. Individuals at the universities and businesses belonging to the two consortiums were approached (by email) for involvement in the study. Only consortium partners not directly involved in the development of PMF platforms were excluded from the study. The sampling frame was chosen to provide a relatively quick overview of the ‘state of play’ in PMF and NPBTs within these two large European projects.

Interviews were audio recorded and transcribed by a private company adhering to the UK Data Protection Act and General Data Protection Regulations of the European Union.

Potential participants were informed of their prospective invitations to these informal interviews during the grant preparation phase. Audio consent was taken prior to the interview. Participants of Pharma-Factory interviews were also invited to correct their transcripts individually for errors and to ensure no commercially or otherwise sensitive data was collected. All project partners were approached for comment on this article prior to submission. Ethical approval for Newcotiana and Pharma-Factory public engagement work package activities was given by the St George’s Research Ethics Committee (refs: SGREC18.0006; SGREC2018.0143).

### Data analysis

The data analysis was undertaken in accordance with *Framework Analysis*, a thematic approach developed by Jane Ritchie and Liz Spencer for large-scale policy work [66]. It is designed for instances in which there are specific questions, a pre-designated sample (often professionals in a given domain), limited timeframes and known *a priori* issues [70]. An initial coding framework was developed by three researchers for both datasets via line-by-line open coding, after which subsequent transcripts were coded and indexed using NVivo 12 for Windows.

The coding and development of analytical themes was conducted separately for each of the studies before a secondary, combined analysis was undertaken by cross-referencing analytical themes and supporting evidence. This approach allowed for exploration of the complementarities and potential differences in the data, which are outlined below. A list of codes and supporting data are provided alongside this article in the project repositories.

### Findings

Between April and May 2018, Jonathan Menary (PhD) and Sara Mesquita de Albuquerque (MSc), both research assistants with experience in qualitative interviewing, conducted 21 separate interviews with participants (17 participants were interviewed by video conferencing or telephone and four were interviewed face-to-face). In total, 51 people were approached for interview. Those that did not respond to requests for interview after two emails were considered to have refused to participate. One participant was present in both samples (due to representation on both projects)– 13 (61.9%; 13/21) were men and seven (33.3%; 7/21) were women. Six participants (28.6%; 6/21) represented SMEs, being businesses with less than 250 employees (one VP, two CEOs and two research scientists), six (28.6%; 6/21) were researchers at universities (three professors, two senior researchers and one project manager) and eight (38% 8/21) were researchers at public research institutions (four professors, three senior researchers and one PhD student). Interviews lasted between 20–40 minutes for Newcotiana and 54–96 minutes for Pharma-Factory. No respondents refused to answer any topic of enquiry that was posed to them.

In this section, the key findings of the research are outlined with reference to three key themes that have emerged. Similar themes were identified in each study, with discussions around NPBTs being somewhat dominant. The findings are presented in a combined manner, but differences between the projects are noted where relevant.

### The need for (careful) communication

For the researchers and companies working in the field of PMF and plant breeding, communication with others—particularly the public—was considered to key to improving the acceptability of the technology:

“… what we need to do is come to the public and explain to the public that… breeding is even worse than genetic modification. I’m not sure that the common regular person in the house is familiar with the real facts.”

Pharma-Factory SME representative (1)

A key finding showed that communicative activities often involved ‘contrast’, most commonly between NPBTs and conventional breeding techniques, which, in the case of mutagenesis, researchers stressed could be far less precise than NPBTs:

“The previous technology was like shotguns. Now we are using a laser to do exactly the same.”

Newcotiana researcher (6)

There was an assumption that the more information the public had about these technologies, the greater would be its support for their development:

“So when you explain to them these kinds of things, they are usually ready to accept such kind of production.”

Pharma-Factory researcher (5)

The same is true for PMF in general, where a need for contrast with existing protein expression systems was perceived to be an advantage for “greener”, plant-based production:

“… [plant molecular farming] is our interest, because getting products coming from plants is like… a green source, so that’s completely okay and complies with our aim in the cosmetic industry.”

Newcotiana cosmetic company representative (1)

Furthermore, effective communication of the benefits of PMF, including the potential lower cost of plant-based expression and the need to focus on the products it can provide, was considered to be important for the future of the technology:

“I’m really, really emphasising making pharmaceuticals, because the alternative is quite expensive.”

Newcotiana researcher (3)

“… we shouldn’t spend so much time on technicalities… this won’t reach out very much to the general public. If you can show that the products that are done by these techniques are interesting, then that might be all that people care about.”

Newcotiana researcher (5)

Although this view contradicts the assumption that providing more information about biotechnology (necessarily) leads to greater support for it, there was a perception that the purpose of PMPs would improve the acceptability of genetic modification:

“…[modification] has a negative picture but if you use it for production of pharmaceuticals then it completely changes.”

Newcotiana researcher (1)

There were reservations amongst several scientists about the extent to which lay people would be able to fully appreciate what were described as “academic” differences between NPBTs and the first-generation GM techniques that have caused controversy in the EU:

“I don’t’ believe that people will [see] a big difference between GMO and new plant breeding techniques. In their minds, it’s more of this whole modern stuff, modern genetic manipulation.”

Newcotiana researcher (5)

One participant described a need for a “blockbuster success” in PMF to increase public awareness of the technology. In providing information about NPBTs and PMF, the media was held to be the most important actor(s), capable of shaping the public’s image of these emerging technologies. It was also suggested that environmental non-governmental organisations (NGOs) would oppose NPBTs, even for biopharmaceutical crops, based on historical responses to GM technology in food:

“One of the main reasons Golden Rice has been sitting on the shelf for ten years is because organisations such as Greenpeace came out and said ‘we cannot allow Golden Rice to go out because if we allow one product the floodgates will open’. As far as Greenpeace is concerned… there is no difference between molecular farming, mutational improvement, agronomic traits, or anything else. They would always, in my opinion, maintain this position—against.”

Pharma-Factory researcher (9)

There was also concern that the combination of genetic modification and the tobacco plant could add to the stigma around an already stigmatised crop for the potential growers of PMF crops. However, many more participants observed that PMF could actively improve the image of the tobacco plant should it be used in the production of pharmaceutical products:

“I think less controversial definitely, definitely. I think that now the uses of tobacco are 99% smoking. That is something that is not good for your health… and the aim of this project is to have a new-cotiana, a new tobacco that changes completely the scope and the uses, instead of being for smoking it’s used for more positive applications.”

Newcotiana researcher (2)

In summary, a strong emphasis was placed on the importance of communication with the public in particular. Engagement of the media was seen to be paramount for enabling positive communications about PMF. Many participants embraced the need for communication with the public by speaking on public radio and at schools. There was a perception that a focus on products—and their benefits—was an important focus for these communications. However, it was also suggested that opposition to the use of NPBTs would persist amongst certain groups regardless of purpose.

### Regulatory environments tied to existing systems and definitions

The technical requirements of PMF are strictly tied to both current regulation and existing protein expression systems. These current systems form a benchmark for quality assessment, yet were also seen as a significant barrier:

*“… it’s really easy and very clear what we need to demonstrate in order to be competitive. You need to demonstrate that you can produce to the same quality and the same quality standards set by for example the pharmaceutical and food industry. It’s quite clear. It’s certain ISO regulations, it’s the GMP* [good manufacturing practice] *regulations*.*”*

PharmaFactory SME representative (3)

“But the CHO cells and bacteria are so established and there are so many examples where processes have been approved by the regulatory agencies… it’s completely different with plants.”

Newcotiana researcher (1)

The lukewarm attitude of big pharmaceutical companies towards new sources of PMPs was likewise noted as a potential barrier to the support for PMF:

“There had been a lot of resistance amongst the big pharmaceutical companies to actually bring on line another production technology because they felt that that would compete with their gold standard, which was transgenic mammalian cells in fermenters… perhaps this is one of the reasons you haven’t seen more than one or two products of plant molecular farming on the market. And still big pharma is, at best, neutral; in a worst case scenario, skeptical or even ambivalent about a plant production system.”

PharmaFactory researcher (9)

Plants were also promoted as a potentially safer platform for the production of PMPs:

“… the product will be safer if it’s produced in plants, because viruses in plants are not infecting humans.”

Pharma-Factory SME representative (5)

It was suggested that the use of controlled environments for PMP production could improve their regulatory standing and, it was also assumed, the social acceptability of genetic modification:

“The major difference is, first GM food is done in the open field, with all the risks that you have. So plant molecular pharmaceutical is in containment… and the other thing is they know they will get sick and they will need medicine.”

Pharma-Factory researcher (4)

If regulatory hurdles can be overcome, then competition from existing systems remains, though it was noted that PMF will compete best in niches for which no alternative protein expression systems exist:

“Because the field of protein production is quite competitive and so far plants only cover a niche within this whole market. And are not really competitive to other systems like microbial cells or mammalian cells.”

Newcotiana researcher (1)

The regulation of NPBTs was also of concern for participants, hinging on the classification of genetic modification:

“In order to make gene editing, you have to introduce CRIPSPR-Cas protein, plus these guide RNA. And one easy way to do it is to make a transgenic plant with the CRISPR-Cas gene, plus the transgenic RNA gene. And that’s transgenic. There is no doubt about it. And then you cross these out and you remain with a mutation. But as the European legislation works by pedigree… anything that has been transgenic once is, according to the European legislation, is transgenic forever. Even, if the transgene is not [there anymore].”

Newcotiana researcher (5)

In summary, there is evidence of both certainty and uncertainty around the regulatory environment. Certain existing systems provide both a benchmark for PMPs and source of direct competition. It is not clear to practitioners whether PMF will be considered for a distinctive regulatory pathway, nor how NPBTs will be regulated in the future.

### Legitimation of new plant breeding techniques

A further conceptual theme that related to the legitimation of NPBTs was identified. Participants pointed to various justifications for their use, such as by appealing to the long history of domestication and cultivation of various crop plants by humans:

“I [would] like [the public] to be able to put it in the context of all the breeding that has been done, is doing, being done and have done for centuries… people, both farmers and general public to understand that this, the changes produced are the same that we have been producing for many years, it’s only the tools we use are different and are more effective.”

Newcotiana researcher (2)

NPBTs were also considered to mimic more “natural” processes, such as gene silencing and knockouts:

“I would say new plant breeding techniques, it’s no different to the natural variation… just making base pair mutations to, for example in my case that knock-down gene. So those single base pair [mutations] will occur, they can occur in nature.”

Newcotiana researcher (3)

In turn, these arguments fed into debates about the appropriate regulatory response by the EU towards NPBTs:

“How should we do it, what should be the risk? How should we be able to regulate such things if you cannot prove this thing is different from a natural plant?”

Newcotiana researcher (6)

In conclusion, the use of NPBTs is being legitimated by (pre-) history, precision and naturalness. Interview themes across both projects overlapped considerably, though more emphasis was placed on products and processes amongst the Pharma-Factory cohort and a greater focus on public perception of NPBTs within the Newcotiana cohort.

## Discussion

In this section, the findings are discussed with reference to the wider literature around plant molecular farming, biotechnology and technological transitions. The results of the study have confirmed several previous findings. The need for plant-specific policies to support PMF, for example, has been noted by other commentators [56]. Some authors have also pointed to a need for blockbuster successes in PMF [64], an observation echoed here. The need for these elements of technological development appears to be well-described by the multi-level perspective, demonstrating its effectiveness at describing the factors that shape the trajectory of new technologies. More surprising are the ways in which NPBTs are being legitimated by experts and, in particular, the extent to which this legitimation is rooted in past debates about plant breeding.

### Public perception of biotechnology and communication

The findings suggest that communication is a vital component for the development of PMF and NPBTs; there appears to be an almost inextricable link between the two at this level. Given the scale of debate about biotechnology in Europe, public perception of the risks and benefits of these new technologies (and their regulation) remains the first major obstacle for NPBT-bred PMPs. The extent to which public opinion about biotechnology currently shape the regulatory stance of EU legislative structures is debatable. However, in the case of GM food, public discourse has had a significant impact on the regulatory process [47] and it is through newspapers, television and the internet that people form their first impressions of new technologies and the risks around them [46,71]. As such, our participants’ concerns over communication of PMF and NPBTs—and the role of the media in mediating early perceptions of new technology—appears to be well-founded.

The sometimes-contradictory views expressed in our findings with respect to *what* and *how* to communicate biotechnology information mirror the intricacy of the relevant literature. The assertion that “more information” about NPBTs would lead to greater acceptance, for example, suggests that some participants subscribe to the knowledge deficit model. As Sturgis & Allum [48] note: “implicit in this programmatic agenda is the claim that ‘to know science is to love it’”. Yet participants themselves also displayed a deficit of knowledge when it comes to research on public attitudes towards science. Most cited knowledge as a major barrier (i.e. the public do not have enough) rather than, for example, negative views towards commercial involvement in scientific studies [52].

As for the concern that the public would not see the “academic” differences between older forms of genetic modification and NPBTs, survey data of European consumers shows that cisgenic breeding is more acceptable than transgenic breeding [72,73], suggesting the public is capable of understanding this distinction. Opinions vary across EU member states, however, and have been shown to be tied more closely to purpose and potential benefit [73]. Connor & Siegrist [51] also find that perceived benefit and risk are more significant determinants of public opinion than knowledge, a claim also made by some participants in this study. As such, providing consumers with *sufficient* and *relevant* information about biotechnology to make informed choices based on potential benefits and risks should be promoted [47]; this view likewise re-orientates the emphasis from (public) knowledge deficits to better science communication and perhaps new forms of public engagement that align with the need for more responsible research and innovation [see 74].

The benefits of PMF are perhaps inherently easier to communicate than those of NPBTs, though taken together third-generation GM crops offer direct benefit to consumers, which Breithaupt [75] argues could improve public perception of biotechnology (and their regulatory standing). The perception of direct personal benefit appears to increase acceptance of genetic modified food crops [76]. Many benefits of first-generation GM crops are found ‘upstream’ of the consumer (herbicide tolerance and insect resistance) rather than in aspects of food quality easily perceptible to the wider public [77]. As Sparrow et al. [78] note: “the early (agricultural) products from large biotechnology companies were mistrusted and directly benefitted farmers but not consumers”. Others see the benefits of GM food accruing to only the large multinational agri-businesses that patent the crops in question [79]. The ability for NPBTs to provide downstream benefits, such as high-value medical molecules, is therefore an important factor for understanding social acceptance now and in the future.

### PMF regulation and the socio-technical regime

In general, participants were explicit about what manufacturing standards needed to be met for new PMF products to be successful (i.e. current GMP guidelines). The advantages of plant-based protein production described by participants are not necessarily being realised yet, due to the need to meet GMP guidelines, which have been developed with bacterial and mammalian cell cultures in mind rather than being inclusive of plants [54,55]. Our findings also suggest that significant prior investment in existing systems by firms may be deterring interest in PMF platforms [63,see 80].

The observation that plants occupy several niches in which they do not directly compete with established protein expression systems is also important; according to the MLP, these niches represent proving grounds for PMF and permit the growth of early “blockbuster successes” [64] that participants believed would be required to raise PMF in the public consciousness.

The findings also confirm a lack of landscape-level pressure on the existing regime. However, certain changes could provide opportunities for PMF to become a more established protein expression system, such as subsidies for PMPs, greater emphasis on quick production times [63] and promotion of PMF as a ‘greener’ sources of medical molecules [81]. These initiatives would also align with the EU’s overarching ‘Bioeconomy Strategy’, which promotes greener sources of industrial products [82]. Yet the ability to produce these molecules rapidly could simply serve to reinforce the regime by providing a fast form of pharmaceutical testing that precedes cell culture batch production [63]. A desire for greener products (as promoted by one participant) could put pressure on existing protein expression systems, but this assumption has several problems: 1) it assumes that consumers are familiar with how pharmaceuticals are currently produced, 2) that PMF is demonstrably more environmentally-friendly than existing bioreactors and 3) it leaves the concept of PMF open to criticism from those who have previously advocated against the diversion of food crops for other uses, as occurred with biofuels in the EU [see 83]. The avoidance of food crops (e.g. using tobacco instead) and the use of containment facilities circumvents this problem, but open-field PMF might be considered non-green where land suitable for food production is used for the production of non-food crops. Other landscape-level pressures that could work against PMF include public acceptance of the technology and the use of NPBTs, as described above.

### Legitimation of NPBTs

The findings demonstrate how experts are legitimating the use of NPBTs. Arguments for the more lenient regulation of NPBTs, for example, rest on their precision and ability to induce changes in plant genomes that are equivalent to natural processes (such as gene silencing); the same arguments are used throughout the literature on NPBTs [84–86]. Parallels were also drawn with existing plant breeding techniques, particularly chemical and radiative mutagenesis, which were described as plant breeding’s “shotgun” to the NPBT “laser”. Of course, an appeal to the precision of NPBTs places emphasis on *process* and not product, despite calls for the use of a product-based regulatory trigger for NPBT-bred plants in Europe [37].

Myskja et al. [87] argue that scientists do not see ‘naturalness’ as relevant to the debate about the ethics of plant breeding; the invocation of naturalness in our sample may be an appeal to the presumed perceptions of a public, that, at least in the EU, does see naturalness as important [72]. The mimicry of natural processes lead Eriksson & Ammann [86] to argue that genome editing can facilitate a transition in perception of what *is* natural in plant breeding by situating it (conceptually) between transgenic techniques and more conventional breeding. NPBTs were also described as simply the most recent of a series of technological advances that connect the Agricultural Revolution (and plant domestication) to plant breeding today.

### PMF with tobacco

In general, the coupling of PMF and NPBTs with the tobacco plant was seen to provide an opportunity to improve the image of tobacco. For US consumers, support for genetically modified tobacco was contingent on societal good [88]–however, given the scale of debate in Europe over the use of GM crops, further research is required to determine European attitudes towards PMF and whether it can genuinely represent a bridge to greater social acceptance of these biotechnologies.

### Limitations and areas for future research

The relative homogeneity of the participants in this study—predominantly (12/21; 57%) researchers at universities and public research institutes—has several limitations. Whilst this is useful for providing an understanding of the views that experts have of the immediate regulatory barriers for PMF and the ways in which experts may or may not have engaged the public, they are not necessarily conversant with the literature on public attitudes towards, for example, genetic modification. Likewise, participants in this study were drawn from two H2020 projects, limiting the scope of the study to Europe. Given differences in legislation and reported measures of public opinion between Europe and the United States, for example, this work cannot be considered applicable to areas outside of Europe. A key area for future research will involve determining the barriers and facilitators of PMF and NPBTs in low- and middle-income countries, given the potential benefits of both to developing nations [see 65]. There is also a need to move towards public engagement activities that bring various stakeholder groups together in order to facilitate two-way communication. The Pharma-Factory project is taking such an approach to the development of PMF (see https://pharmafactory.org).

## Conclusions

This study has outlined an approach to identifying the barriers and facilitators for scaling-up PMF and NPBTs using an applied qualitative framework. It has identified public perceptions of biotechnology, the existing regulatory environment and competition from other protein expression systems as explicit barriers to scaling-up PMF and NPBTs. This study has sought to contextualise these insights within the wider literature around PMF and NPBTs and pointed towards useful conceptual frameworks—such as MLP—that could help researchers understand the development of PMF in Europe. In particular, the lack of plant-specific regulation, landscape-level pressure on the existing regime of bacterial and mammalian protein expression systems and support from pharmaceutical companies may confine PMF to ‘niches’ for the foreseeable future.

This article has described a number of possible facilitation mechanisms, such as science communication focused on the benefits and purpose of PMF, which our participants identified as important in leading to an improvement in the perception of genetic modification of plants amongst the public. The study has also shown how scientists and other interested parties are framing NPBTs to legitimate more favourable regulation, relying primarily on arguments of precision, mimicry of natural processes and plant breeding heritage.

Lessons of the past can yield important insights into the way forward through current challenges. The main challenges at the intersection of PMF and NPBTs will likely be regulation and the opinions of Europeans towards these technologies. As our participants have stated—and has been realised in the past (e.g. GM crops)–these two topics are central to the scale-up of new technologies. The top-down promotion of science and technology for its own sake has not always been persuasive and, as the findings of this study indicate, scientists do not always appear to be aware of the wider literature on acceptability. As such, there is a need for sufficient and relevant information about new technologies alongside more active forms of public engagement that link both researchers and other stakeholders. Hartley et al. [89] argue that new agricultural biotechnology represents an opportunity to re-evaluate current governance systems—it is hoped that our work can contribute to the socially-responsible governance of the emerging fields of PMF and NPBTs.

## Abbreviations

CHO: Chinese hamster ovary
CRISPR: Clustered regularly interspaced palindromic repeats
CSIC: Consejo Superior Investigaciones Científicas
ECJ: European Court of Justice
EU: European Union
GM: Genetically modified
GMP: Good manufacturing practice
ISO: International Organization for Standardization
MLP: Multi-level perspective
NPBT: New plant breeding technique
PMF: Plant molecular farming
PMP: Plant-made pharmaceutical
SME: Small to medium enterprises
